# Meta-analysis of gut microbiota alterations in patients with irritable bowel syndrome

**DOI:** 10.3389/fmicb.2024.1492349

**Published:** 2024-12-24

**Authors:** Xiaxi Li, Xiaoling Li, Haowei Xiao, Jiaying Xu, Jianquan He, Chuanxing Xiao, Bangzhou Zhang, Man Cao, Wenxin Hong

**Affiliations:** ^1^Department of Gastroenterology, Shenzhen Hospital, Southern Medical University, Shenzhen, China; ^2^College of Rehabilitation Medicine, Fujian University of Traditional Chinese Medicine, Fuzhou, China; ^3^Center for Research and Development, Xiamen Treatgut Biotechnology Co., Ltd., Xiamen, China; ^4^Department of Rehabilitation, School of Medicine, Zhongshan Hospital of Xiamen University, Xiamen University, Xiamen, China; ^5^College of Pharmacy, Fujian University of Traditional Chinese Medicine, Fuzhou, China; ^6^Department of Gastroenterology, The Second Affiliated Hospital of Fujian University of Traditional Chinese Medicine, Fuzhou, China

**Keywords:** irritable bowel syndrome, gut microbiota, meta-analysis, random forest model, transfer learning

## Abstract

**Introduction:**

Irritable bowel syndrome (IBS) is a common chronic disorder of gastrointestinal function with a high prevalence worldwide. Due to its complex pathogenesis and heterogeneity, there is urrently no consensus in IBS research.

**Methods:**

We collected and uniformly reanalyzed 1167 fecal 16S rRNA gene sequencing samples (623 from IBS patients and 544 from healthy subjects) from 9 studies. Using both a random effects (RE) model and a fixed effects (FE) model, we calculated the odds ratios for metrics including bacterial alpha diversity, beta diversity, common genera and pathways between the IBS and control groups.

**Results:**

Significantly lower alpha-diversity indexes were observed in IBS patients by random effects model. Twenty-six bacterial genera and twelve predicted pathways were identified with significant odds ratios and classification potentials for IBS patients. Based on these feature, we used transfer learning to enhance the predictive capabilities of our model, which improved model performance by approximately 10%. Moreover, through correlation network analysis, we found that Ruminococcaceae and Christensenellaceae were negatively correlated with vitamin B6 metabolism, which was decreased in the patients with IBS. Ruminococcaceae was also negatively correlated with tyrosine metabolism, which was decreased in the patients with IBS.

**Discussion:**

This study revealed the dysbiosis of fecal bacterial diversity, composition, and predicted pathways of patients with IBS by meta-analysis and identified universal biomarkers for IBS prediction and therapeutic targets.

## 1 Introduction

Irritable bowel syndrome (IBS) is a prototypical psychosomatic condition of the digestive system, closely related to psychological, social, and environmental factors (Ng et al., 2024). It is a disorder of the brain–gut axis characterized by frequent abdominal pain, bloating, flatulence, and changes in bowel habits—either constipation or diarrhea—which has an impact on quality of life compared to inflammatory bowel disease (IBD). The global prevalence of IBS is 4% but varies across different regions (Oka et al., 2020), and the prevalence of IBS is higher among women than men (Sperber et al., 2021). There is still a lack of accurate diagnostic criteria for IBS, with no detectable anatomical, inflammatory, or biochemical pathology, and it is defined based on symptom criteria, including recurrent abdominal pain associated with changes in bowel habits. According to the Rome IV diagnostic criteria, IBS is classified into four types: IBS with predominant constipation (IBS-C), IBS with predominant diarrhea (IBS-D), IBS with mixed bowel habits (IBS-M), and IBS unclassified (IBS-U).

Globally, the incidence and prevalence of IBS are relatively high, resulting in increased medical costs and healthcare expenses (Zhen et al., 2006; Ma et al., 2021). The specific cause of the syndrome remains uncertain, despite being described more than a 100 years ago. Several factors and mechanisms may play a role in the pathogenesis of the syndrome (Chang and Talley, 2011). These include altered gastrointestinal motility, visceral hypersensitivity, post-infectious reactivity, brain–gut interactions, alteration in the fecal microbiome, bacterial overgrowth, food sensitivity, carbohydrate malabsorption, and intestinal inflammation, and all of these indicators have been studied as mechanisms involved in the pathogenesis of IBS (Occhipinti and Smith, 2012; Ng et al., 2018). However, the diagnosis and treatment of IBS still pose challenges in clinical practice. Meanwhile, due to the physiological interactions between humans and their microbiome, many diseases are hypothesized to be associated with alterations in the “healthy” gut microbiota. These include metabolic disorders, inflammation and autoimmune diseases, neurological disorders, and cancer (Zhu et al., 2013; Turnbaugh et al., 2006; Son et al., 2015; Wang et al., 2012; Scheperjans et al., 2015). Certain gut-related diseases such as obesity and IBD have been extensively studied in human cohorts and animal experiments, revealing significant and sometimes causal associations with the microbiota (Walters et al., 2014). Gut microbe-induced immunomodulation strategies for therapeutic intervention of inflammatory diseases may be effective. Moreover, released SCFAs can potentially suppress the Th1 response by inhibiting the activation of nuclear factor kappa B (NF-kB), thereby reducing the production of inflammatory cytokines (Mukherjee et al., 2018). Previous studies have stimulated research on many complex diseases with unknown etiology, which are suspected to be related to the microbiome (Kassinen et al., 2007; Krogius-Kurikka et al., 2009; Carroll et al., 2012; Rajilić–Stojanović et al., 2011; Jeffery et al., 2012). Increasing evidence highlights the crucial role of the gut microbiota in both health and IBS.

Some studies on the composition of the microbiota using 16S rRNA gene sequencing or shotgun metagenomics have shown that, in some cases, there is minimal association between changes in diversity and taxonomy with IBS (Labus et al., 2017; Tap et al., 2017; Jeffery et al., 2020). Other studies have reported reduced diversity and taxonomic alterations, but these findings are inconsistent across the research (Durbán et al., 2012; Lo Presti et al., 2019). Recent research has shifted toward assessing the functional characterization of the microbiota through shotgun metagenomics or metabolomics (evaluating bacterial metabolites detected in feces), with the former assessing functional potential through microbial gene content. For instance, Mars et al. combined multi-omics data from the gut microbiome, metabolome, host epigenome, and transcriptome in IBS, identifying subtype-specific and symptom-related microbial changes, such as purine metabolism (Mars et al., 2020). Su et al. compared the taxonomic and functional composition of the gut microbiota among 942 participants with IBS-D, IBS-C, and IBS-U and 942 non-IBS controls, based on 16S sequencing data. They found that, compared to participants with IBS-C, those with IBS-D or IBS-U exhibited significantly reduced bacterial diversity. Distinct bacterial signatures were associated with different IBS subtypes, and the related functional changes were relevant to the pathogenesis of IBS (Su et al., 2023). Jacobs et al. collected fecal samples from 318 patients with IBS and 177 healthy controls for 16S rRNA sequencing and found that IBS is associated with alterations in microbial community functions, such as a significant increase in the abundance of *Bacteroides* in patients with IBS (Jacobs et al., 2023). Although these studies have reported significant changes in gene content and metabolites, the specific features vary across studies. Overall, unlike other gastrointestinal diseases, such as IBD, there has not been a strong functional microbial signature consistently associated with IBS diagnosis.

To further understand the functional characteristics of the gut microbiota in IBS, we collected and analyzed a dataset consisting of 1,167 samples, including 623 patients with IBS and 544 healthy controls without gastrointestinal diseases. This dataset was used for a meta-analysis of the gut microbiota, including compositional assessment through 16S rRNA gene sequencing and functional assessment. Modeling analysis was performed based on the aforementioned results, resulting in a well-performing machine learning model for discrimination (Gong et al., 2022; Wirbel et al., 2021). We also utilized transfer learning models to achieve better results on other datasets (Xu et al., 2023).

## 2 Methods

### 2.1 Literature search and study selection

Based on the Preferred Reporting Items for Systematic Reviews and Meta-Analyses (PRISMA) standard (Page et al., 2021), the following keywords were selected to search the literature included in the NCBI PubMed databases before December 2022: (microorganism OR microbe OR germ OR microbiome OR microbiota OR gut OR intestinal) AND (feces OR fece OR stool) AND (irritable bowel syndrome OR IBS).

**Figure 1:**
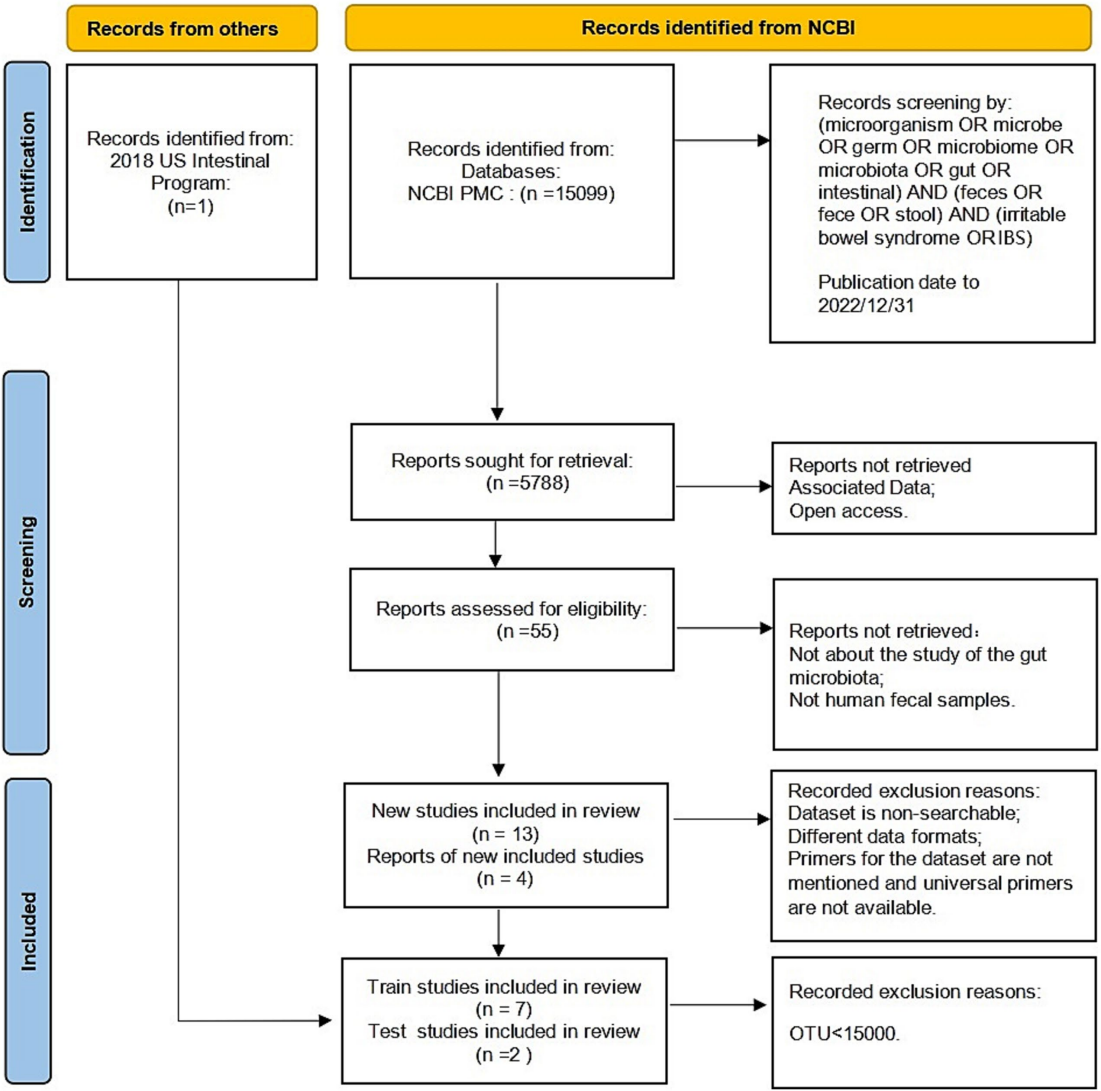
Description of the selection of the included studies following a PRISMA flow diagram.

The raw data and metadata for the included cohorts in the study were downloaded from the Sequence Read Archive (SRA), a public repository for sequencing data. The sequencing methods used in these studies included Illumina sequencing and 454 sequencing. We excluded studies that focused on culture and qPCR techniques or were only abstracts from conference papers. Studies without controls or with fewer than five patients were also excluded. In addition, any studies that did not provide publicly available sequences or metadata were excluded. The reuse of these published data in our meta-analysis adhered to all relevant ethical regulations. Finally, we collected 1,167 fecal samples from seven previously published studies (Zhuang et al., 2018; Belkova et al., 2020; Zhu et al., 2019; Liu et al., 2020; Pozuelo et al., 2015; McDonald et al., 2018; Vork et al., 2021). Figure 1 displays the detailed status of the data collection.

### 2.2 Data processing

The raw sequence data and metadata were obtained from the Sequence Read Archive (SRA) at the NCBI. Each dataset was imported and assembled in Unoise3 as single-end or paired-end reads for either 454 or Illumina sequencing (Edgar, 2016). To avoid biases introduced by different bioinformatics analysis pipelines, the raw sequence data were preprocessed. Clean, high-quality reads were obtained through sequence merging and quality control. First, when Fast Length Adjustment of SHort Reads (FLASH V1.2.11) was used to assemble the paired-end reads for the V3-4 region (Magoč and Salzberg, 2011), the -x 0.15 option was selected to control the maximum mismatched base pairs ratio in the overlap area, and the -M 150 or -M 250 option was selected to control the maximum length of the overlap area. Then, cutadapt (V1.13) was used to trim and filter the sequence data processed by FLASH (Martin, 2011), including removing adapter sequences and discarding sequences with fewer than the specified number of bases. Denoising was performed using Unoise3, an algorithm that generates zero-radius operational taxonomic units (zOTUs) by directly denoising without clustering. Subsequently, the resulting representative sequence set was aligned and classified using the SILVA database (silva_132_97). Samples with less than 15,000 zOTUs were discarded. Using the 16S rRNA gene sequencing data and Kyoto Encyclopedia of Genes and Genomes (KEGG) orthology, we performed Phylogenetic Investigation of Communities by Reconstruction of Unobserved States (PICRUSt2) analysis for the functional prediction of the microbiota in the intestine (Douglas et al., 2020). The America dataset contains a large number of samples, so the samples were split into two datasets based on nationality, and the healthy controls were matched 1:1 based on the information about the patients with IBS. The comprehensive details of the datasets are presented in Table 1.

**Table 1:** Characteristics of the datasets included in the fecal sample-based analysis with zOTUs.

Source	Country	HS	IBS	Region(s)	Sequencing platform	Library Layout
PRJNA475187 (Zhuang et al., 2018)	China	9	20	V3-V4	Illumina Miseq	Paired
PRJNA604466 (Belkova et al., 2020)	Russia	43	10	V3-V4	Illumina HiSeq	Paired
PRJNA566284 (Zhu et al., 2019)	China	14	15	V4	Illumina Miseq	Paired
PRJNA544721 (Liu et al., 2020)	China	44	84	V3-V4	Illumina Miseq	Single
PRJNA268708 (Pozuelo et al., 2015)	Spain	66	125	V4	Illumina Miseq	Single
PRJEB11419USA (McDonald et al., 2018)	United States	102	102	V4	Illumina Miseq	Single
PRJEB11419UK (McDonald et al., 2018)	United Kingdom	146	146	V4	Illumina Miseq	Single
PRJNA682378 (Vork et al., 2021)	China	78	71	V4	Illumina MiSeq	Single
PRJNA1011519	China	65	85	V4	Illumina Miseq	Paired

### 2.3 Community analysis

Based on the OTU tables derived from each study, alpha diversity analysis, beta diversity analysis, and species composition analysis were performed within each dataset. Alpha diversity indices (such as evenness, observed OTUs, and Shannon Index) were analyzed using the Wilcoxon test (Segata et al., 2011; Thomas et al., 2019; Wu et al., 2021). Principal coordinates analysis (PCoA) based on the Bray–Curtis distance at the genus level was used for beta diversity to visualize the differences in the microbial community structure across the samples. Significance tests for beta diversity indices were conducted using permutational multivariate analysis of variance (PERMANOVA) with 10^4 permutations in the vegan R package (Oksanen et al., 2013). Finally, a meta-analysis of the bacterial alpha diversity and beta diversity indices, microbial taxa, and pathways across the seven studies was performed using the “metafor” package (version 2.4–0) to assess consistency using both a random effects (RE) model and the fixed effects (FE) model (Viechtbauer, 2010). The analysis was performed in the R environment (version3.6.3). The co-occurrence network of the microbes was constructed with the Pearson correlation coefficient. The topological coefficients of the network were calculated using the R package” igraph.”

### 2.4 Model construction and evaluation

With the aim of investigating the potential utility and generalizability of the distinctive genera and KO makers in effectively discriminating between individuals diagnosed with IBS and HS within a classification model, we partitioned our dataset into training and test sets in a 7:3 ratio. We applied the traditional random forest in study-to-study transfer validation. In addition, we employed data from the seven studies to meticulously construct our classification model. Transfer learning involves leveraging existing knowledge to acquire new insights (Xu et al., 2023), with its essence lying in identifying commonalities between established and novel knowledge domains. Through transfer learning, an appropriate model can be adapted to a new task by considering the similarities and differences between different tasks, and by adopting adaptive learning, the model can be flexibly adjusted to meet the needs of different tasks. Given the inherent variability in samples across diverse studies, attempting to enhance accuracy by applying models from one study to test data from another is not straightforward. We employed the area under curve (AUC) metric to evaluate the performance of the model and constructed a receiver operating characteristic (ROC) curve based on the true positive rate (TPR) and the false positive rate (FPR). Meanwhile, we conducted a comparative analysis between the validation outcomes derived from the transferred model and those obtained from the original, non-transferred model. This comparative assessment enabled us to effectively gage the potency of the transferred model.

## 3 Results

### 3.1 The community diversity analysis of IBS and HS

**Figure 2:**
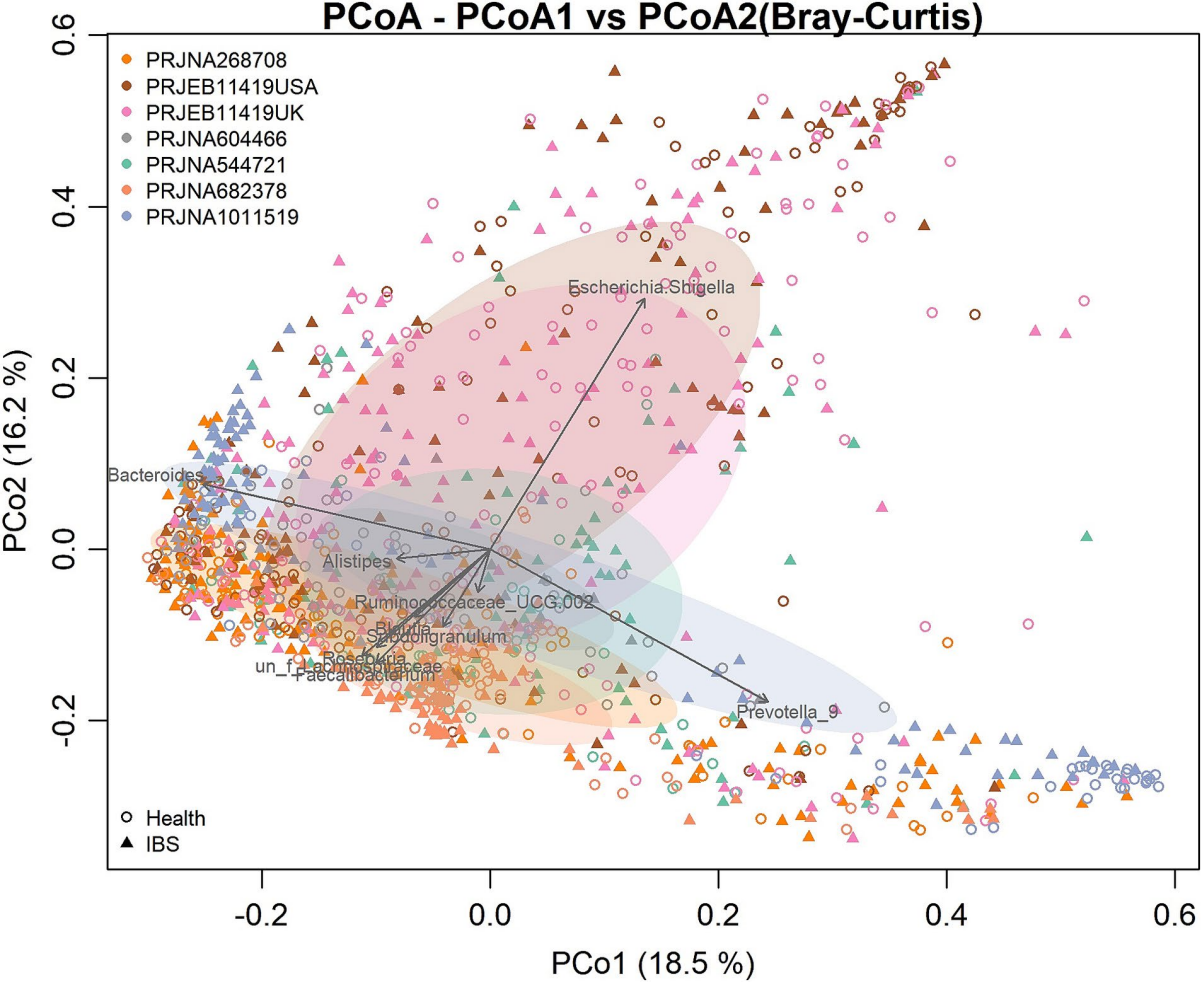
Principal coordinates analysis (PCoA) based on the Bray–Curtis distance according to the genera. Each point in the diagram represents a sample. The shapes represent the healthy control and IBS groups, respectively. The colors represent the different studies.

There were significant differences in the overall microbial community structure among all groups when all samples from the seven studies were combined (PERMANOVA, *F* = 6.25, *p* = 0.001). However, the PCoA plot based on the Bray–Curtis distance showed that the samples were clustered mainly by the individual studies, which may be attributed to the differences in the sample populations, DNA extraction methods, sequencing regions of the 16S rRNA gene, and sequencing platforms used by the individual studies (Figure 2). To more objectively reflect the consistent differences in the gut bacterial community between the IBS and control groups, we performed a meta-analysis on the microbial metrics from each individual study in the following analysis.

**Figure 3:**
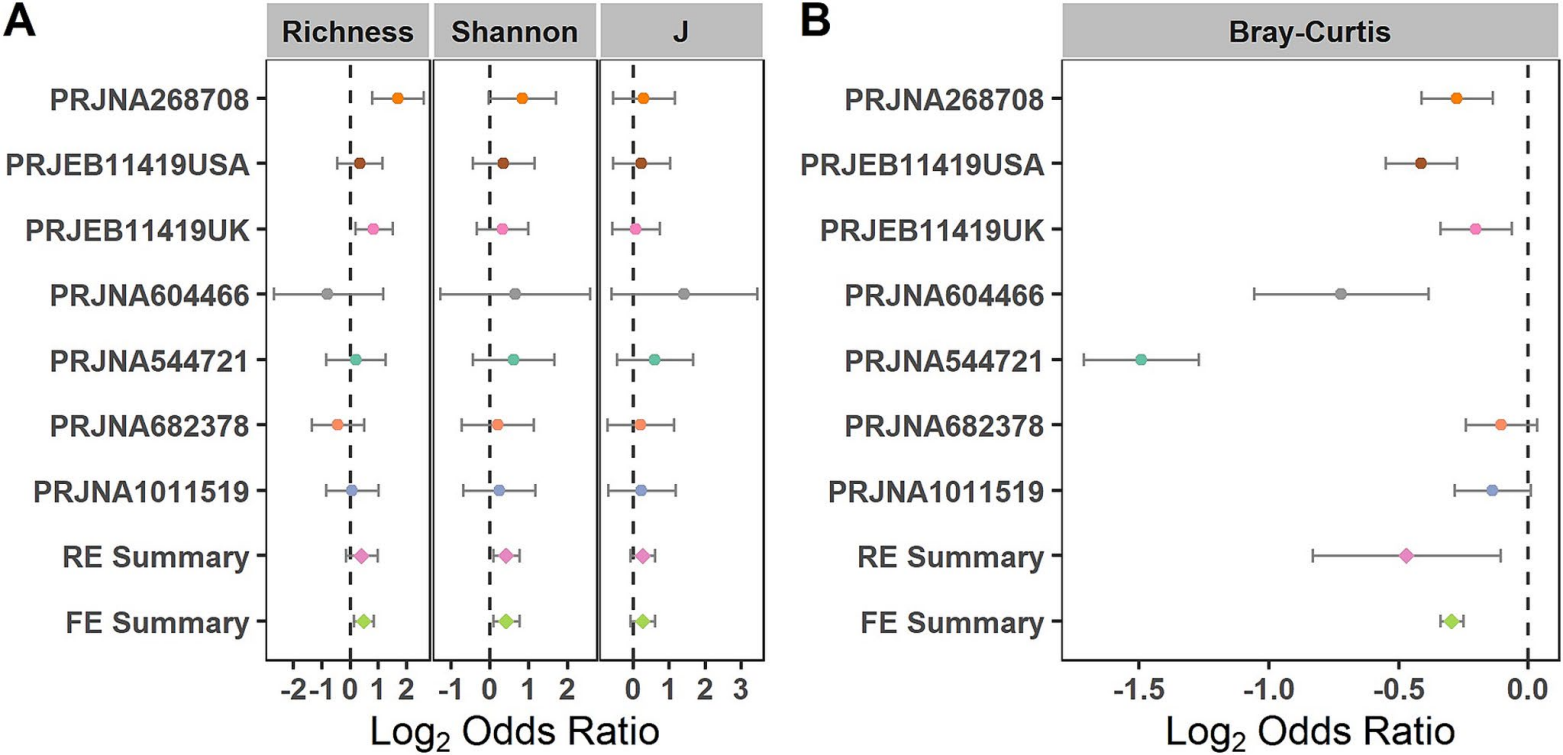
Comparison of bacterial alpha diversity and beta diversity between the individuals with IBS and HS. **(A)** Forest plot of the alpha diversity metrics richness, Shannon Index, and evenness (J) between the individuals with IBS and HS; **(B)** Forest plot of the Bray–Curtis distance between the individuals with IBS and HS. The error bar depicts the 95% confidence interval. The ORs less than 1.0 (left side of the dashed line) indicate that the metric was higher in the patients with IBS than in the controls. The ORs greater than 1.0 (right side of the dashed lines) indicate that the metric was lower in the patients with IBS than in the controls. No intersection between the dashed line and the error bar indicates a significant difference between the patients with IBS and the controls.

We evaluated the differences in the alpha diversity metrics between the healthy controls and patients with patients at the zOTU level. For the alpha diversity metrics, evenness, richness, and Shannon Index were calculated. The Shannon Index showed significant odds ratios (ORs) greater than 1.0 (Figure 3A), indicating that this index in the control group was significantly higher than that in the IBS group. When compared in the individual studies, two of the seven studies (PRJNA268708 and PRJEB11419UK) observed significantly higher microbial richness in the healthy controls than in the IBS group (Supplementary Table S1).

When evaluating the differences in the entire bacterial community between the IBS and control groups using PERMANOVA at the zOTU level, significant differences in the overall communities between the IBS and control groups were obtained in six of the seven studies (Supplementary Table S2) (PRJNA268708, PRJEB11419UK, PRJNA604466, PRJNA544721, PRJNA682378, and PRJNA1011519). Using the RE model, significant bacterial community differences were observed between the IBS and control groups (Figure 3B). When compared in the individual studies, five of the seven studies (PRJNA604466, PRJNA544721, PRJNA268708, PRJEB11419USA, and PRJEB11419UK) observed significant bacterial community differences between the IBS and control groups (Figure 3B).

### 3.2 Identification of cross-cohort species biomarkers for IBS

**Figure 4:**
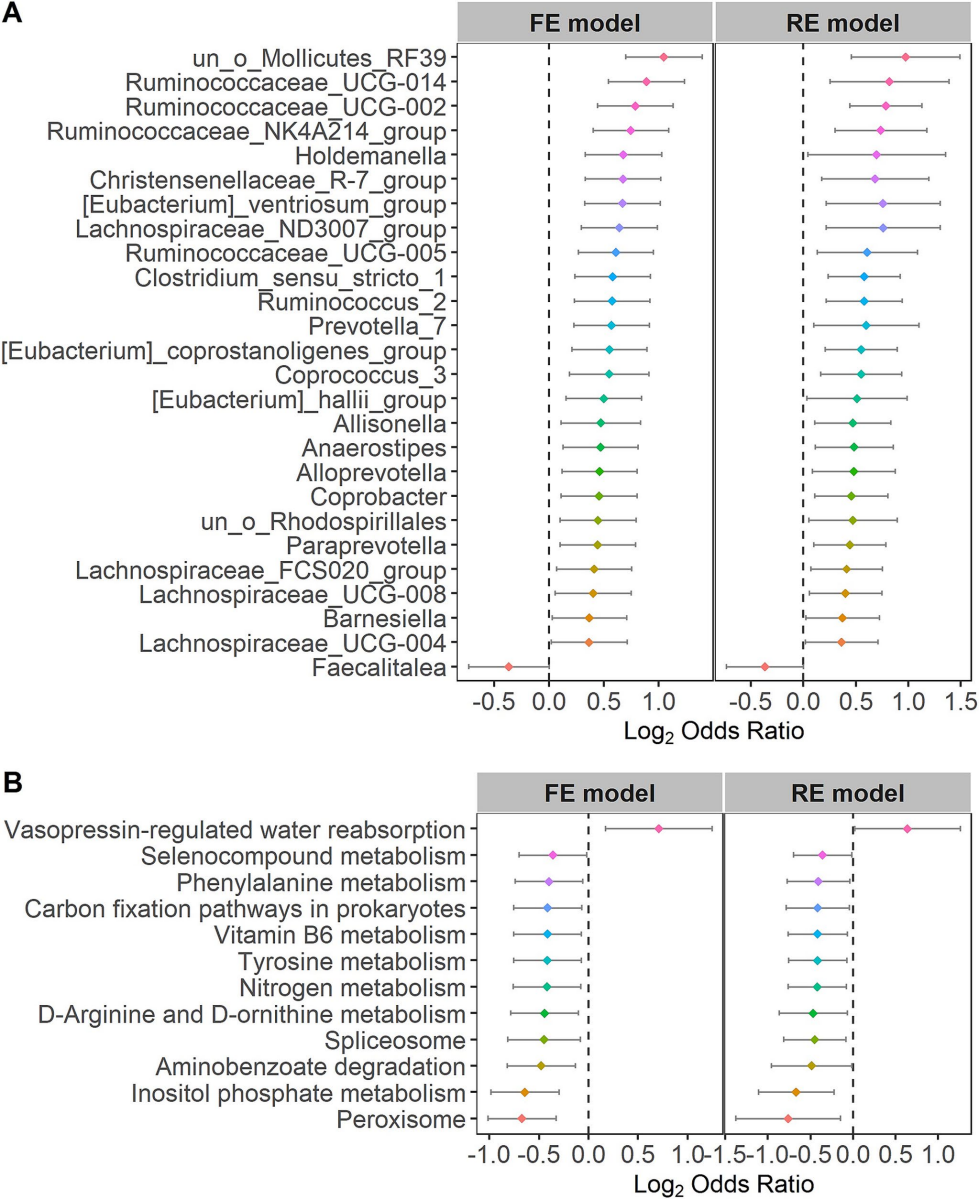
Forest plots of **(A)** the genera or **(B)** the KOs with the significant ORs.

For the purpose of further identifying the significantly different taxa and bacterial pathways between the control and IBS groups, we calculated the ORs of all common taxa and pathways in each study. We identified 26 genera and 12 pathways that were significantly associated with IBS. *Faecalitalea* had significant ORs lower than 1.0 for the IBS group in the RE models. The 26 genera, including *Lachnospiraceae*, *Ruminococcaceae*, *Holdemanella*, *Christensenellaceae*, *Eubacterium*, *Clostridium*, *Ruminococcus*, *Prevotella*, *Coprococcus*, *Allisonella*, *Anaerostipes*, *Alloprevotella*, *Coprobacter*, *Paraprevotella* and *Barnesiella*, had significant ORs higher than 1.0 for the individuals in the control group (Figure 4A), indicating that these bacteria were scarce in the patients with IBS. Vasopressin-regulated water reabsorption had significant ORs higher than 1.0 for the individuals in the control group in the RE models. The 11 pathways, including peroxisome, inositol phosphate metabolism, aminobenzoate degradation, spliceosome, D-arginine and D-ornithine metabolism, nitrogen metabolism, tyrosine metabolism, vitamin B6 metabolism, carbon fixation pathways in prokaryotes, phenylalanine metabolism, and selenocompound metabolism, had significant ORs lower than 1.0 for the individuals in the IBS group (Figure 4B). The statistical analysis of the microbial community in the two groups is presented in Supplementary Table S3. The significant differences at other species levels are shown in Supplementary Figures S1, S2.

### 3.3 Predictive performance of the model on IBS and HS

**Figure 5:**
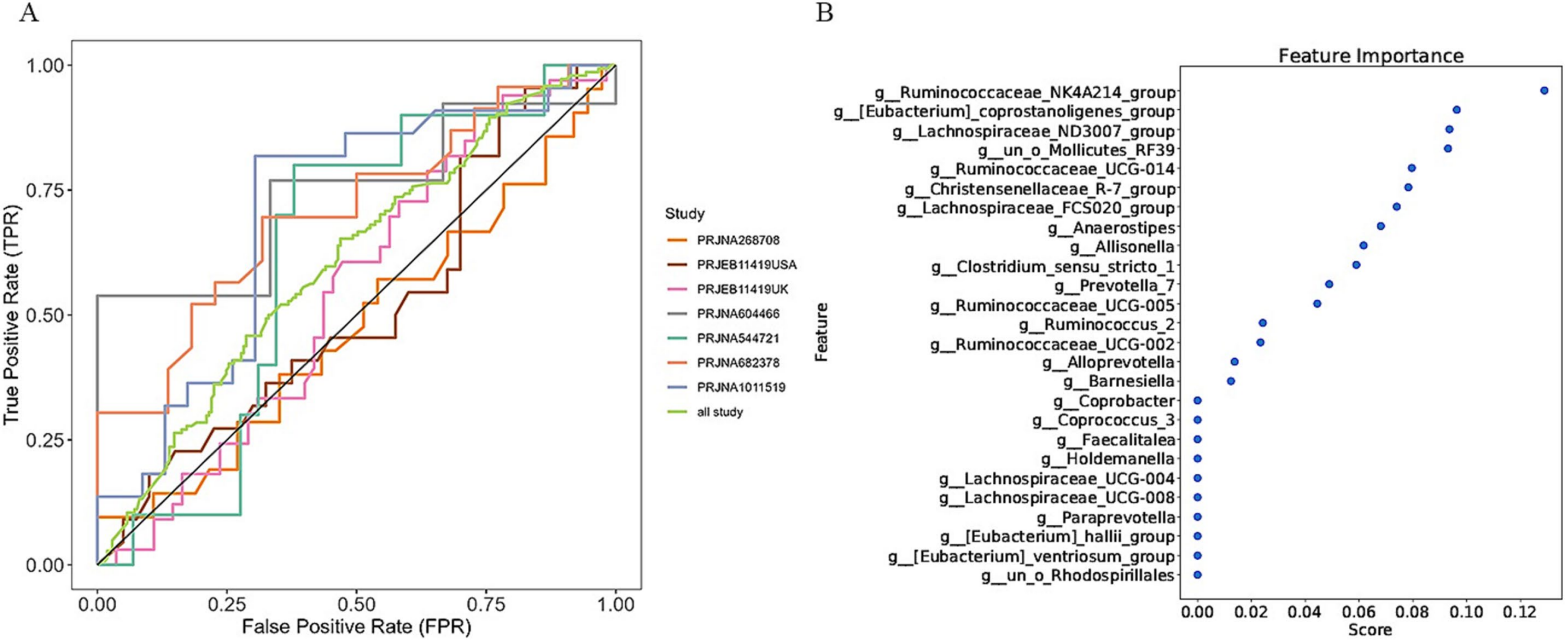
Establishment of an IBS discriminative model based on the abundance of the bacterial genera. **(A)** Diagnostic potential of intestinal bacteria for IBS across all studies. The specific AUC values for the training set and test set are shown in Table 2; **(B)** The rank of feature importance for the optimal model.

**Figure 6:**
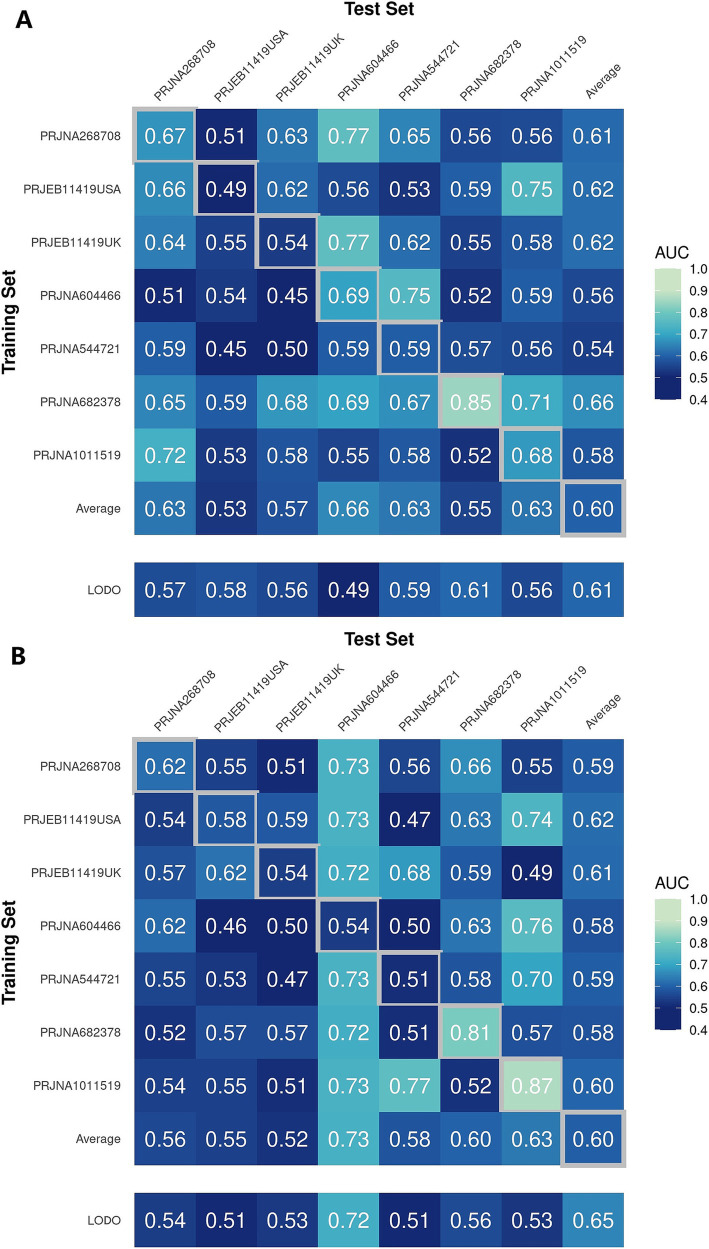
Cross-prediction matrix reporting the prediction performances as the AUC values obtained using a random forest (RF) model on the **(A)** genus and **(B)** pathway level-relative abundances. The values boxed in yellow squares on the diagonal are the AUC values obtained by training and validating within the individual cohorts. The non-diagonal values refer to training a classifier on the dataset corresponding to the row and applying it to the dataset corresponding to the column to obtain the AUC values. The row averages are the average values excluding the diagonal values.

In a previous study, we identified a total of 26 different genera and 12 KOs. These identified genera/KOs have been used as pivotal features in our model construction endeavors, contributing to the development of their corresponding models. The comprehensive outcomes across the various studies, as well as the amalgamated findings encompassing all examined studies, are meticulously presented in Tables 2, 3. As shown in Figure 5A, the test set result of the overall study at the genus level was 0.6384, and the test set result of the overall study at the pathway level was 0.6074. Meanwhile, we present the contribution ranking of the features in the model (Figure 5B; Supplementary Figure S3). We used the above-mentioned bacterial genera to train models on the respective training sets of each dataset and then validated them on the validation sets of each dataset separately. We achieved an average internal AUC of 0.6443, with a range of 0.49–0.85 along the diagonal. The average AUC of the test sets did not reach 0.7, and only four training sets had an average AUC higher than 0.6 (Figure 6A). The classifier had an average AUC of 0.6 (off-diagonal column average), and all models had an average external AUC higher than 0.54 (off-diagonal row average), indicating its effectiveness across the different cohorts.

**Table 2:** The results of the different studies at the genus level.

Dataset	Training AUC	Testing AUC
PRJNA268708	0.85202	0.67310
PRJEB11419USA	0.90171	0.49205
PRJEB11419UK	0.79621	0.53774
PRJNA604466	0.99524	0.69231
PRJNA544721	0.86390	0.59310
PRJNA682378	0.96011	0.84585
PRJNA1011519	0.93417	0.67894
All studies	0.71942	0.63841

**Table 3:** The results of the different studies at the pathway level.

Dataset	Training AUC	Testing AUC
PRJNA268708	0.81136	0.62033
PRJEB11419USA	0.80060	0.58466
PRJEB11419UK	0.76986	0.54077
PRJNA604466	0.99285	0.53846
PRJNA544721	0.79759	0.51379
PRJNA682378	0.91596	0.81126
PRJNA1011519	0.94599	0.86759
All studies	0.64749	0.60742

In addition, when the models were trained at the KO functional prediction level, the classifier had an average AUC of 0.6 (Figure 6B; off-diagonal column average), and all models had an average external AUC higher than 0.58 (off-diagonal row average), indicating its effectiveness across the different cohorts. When validating on the different datasets’ test sets, the average AUC of the test sets did not reach 0.7, and only three test sets had an average AUC higher than 0.6, raising the external mean AUC from 0.54 to 0.63 (Figure 6B). The features closely related to the patients with IBS and healthy controls did not show the expected effectiveness when training models to be used across datasets. The insights gleaned from the depicted figure revealed a discernible pattern: the features selected through meta-analysis can play a role in the modeling process, which helps distinguish IBS and HS.

### 3.4 Performance of transfer learning models on IBS and HS

From the outcomes derived from the aforementioned modeling endeavors, it is evident that superior results cannot be achieved when examining individual studies in isolation. In light of this, we used transfer learning to enhance the predictive capabilities of our model. After comprehensively considering the results of the different studies, it was found that the overall results of the PRJNA682378 study were better. Therefore, the PRJNA682378 model was used as a suitable transfer candidate model. Subsequently, transfer learning was performed on the different studies at the genus level. The results of both pre-transfer and post-transfer are shown in Table 4; Supplementary Figure S4A. In addition, the results at the pathway level are shown in Supplementary Table S4 and Supplementary Figure S4B. It can be seen that after transfer learning, the results of the majority of the studies improved. Although the results of the individual studies did not improve, they were basically the same as the results before transfer learning. Overall, the use of transfer learning can improve the performance of the model.

**Table 4:** Pre-transfer learning and post-transfer learning results at the genus level.

Dataset	Post-AUC	Pre-AUC
PRJNA268708	**0.70399**	0.67310
PRJEB11419USA	**0.54205**	0.49205
PRJEB11419UK	**0.60331**	0.53774
PRJNA604466	0.61538	0.69231
PRJNA544721	**0.62414**	0.59310
PRJNA1011519	**0.78854**	0.67894

Bolded fonts indicate an improvement in performance compared to before transfer learning.

### 3.5 Co-occurrence network for the patients with IBS

**Figure 7:**
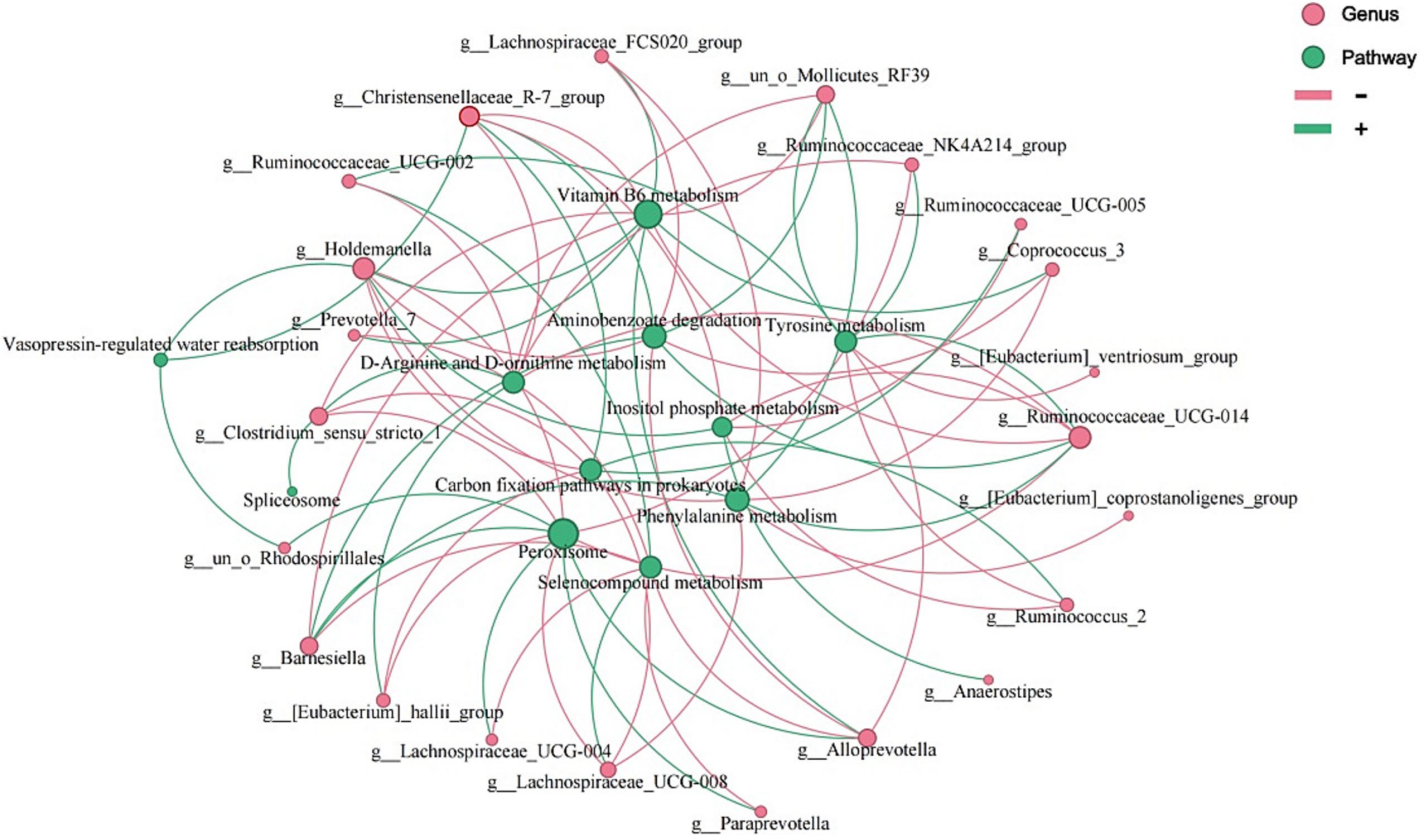
Co-occurrence network plot showing the correlations between the 12 pathways and 26 genera that had significant Spearman correlation coefficients (i.e., with |correlation coefficient | > 0.5 and *p* < 0.05). The size of the nodes varies with the number of edges (red lines, negative correlations; green lines, positive correlations).

We quantified the relationships between the 26 different genera at the genus taxonomic level and the 12 KOs at the pathway level in the patients with IBS using the Pearson correlation coefficient. *Ruminococcaceae* and *Christensenellaceae* were increased in the patients with IBS (Figure 4A). Moreover, *Ruminococcaceae* and *Christensenellaceae* were negatively correlated with vitamin B6 metabolism (Figure 7), which was decreased in the patients with IBS (Figure 4B). *Ruminococcaceae* was also negatively correlated with tyrosine metabolism (Figure 7), which was decreased in the patients with IBS (Figure 4B).

### 3.6 The interconnection of the identification of the cross-cohort species biomarkers

We observed that the abundance of *Ruminococcaceae*, *Anaerostipes*, and *Christensenellaceae* was increased in the patients with IBS in our study. The latest study reported that *Ruminococcaceae* was positively associated with IBS (Tana et al., 2010). Another study reported that *Anaerostipes* was increased in IBS-C (Chassard et al., 2012). A higher relative abundance of *Christensenellaceae* in healthy controls compared to individuals with IBS was reported in several studies (Pozuelo et al., 2015; Jalanka-Tuovinen et al., 2014; De Palma et al., 2017; Hollister et al., 2020). Several studies also reported a positive correlation between *Christensenellaceae* and longer transit time or even constipation (De Palma et al., 2017; Oki et al., 2016; Roager et al., 2016; Tigchelaar et al., 2016; Jalanka et al., 2019).

Tyrosine metabolism, vitamin B6, and phenethylamine were clustered in the patients with IBS. Rearranged during transfection (RET) is a neuronal growth factor receptor tyrosine kinase critical for the development of the enteric nervous system (ENS), which may lead to the hyperinnervation of visceral afferent neurons in the GI tract and contribute to the pathophysiology of IBS (Schenck Eidam et al., 2018). It may become a target for treating IBS (Schenck Eidam et al., 2018). Low intake of vitamin B6 is associated with irritable bowel syndrome symptoms (Ligaarden and Farup, 2011). A previous study reported that an anaerobic, Gram-positive, and IBS-associated bacterium, *Ruminococcus gnavus*, produces tryptamine and phenethylamine by utilizing the dietary amino acid tryptophan and phenylalanine to induce diarrheal symptoms in patients with IBS (Zhai et al., 2023).

*Ruminococcaceae* and *Christensenellaceae*, which were positively associated with IBS (Pozuelo et al., 2015; Tana et al., 2010; Jalanka-Tuovinen et al., 2014; De Palma et al., 2017; Hollister et al., 2020), were negatively correlated with vitamin B6 metabolism in the patients with IBS. We are also aware that low intake of vitamin B6 is associated with IBS (Ligaarden and Farup, 2011). Tyrosine metabolism contributed to the pathophysiology of IBS, while it was negatively correlated with *Ruminococcaceae*. Therefore, these results reveal how the gut microbiota of patients with IBS affects the disease through metabolic pathways.

## 4 Discussion

The study aimed to identify universal gut microbiota biomarkers for IBS prediction and therapeutic targets. We employed a consistent workflow to process raw data, minimizing technical variations between studies. Through the dataset of 1,167 samples, we addressed whether the microbial community of patients with IBS differs from healthy individuals. Overall, the study identified universal biomarkers for IBS prediction and therapeutic targets. The RF model can help choose suitable bacterial strains for precision medicine, benefiting patients with IBS. Transfer learning can assist in transferring the performance of a well-performing model to other datasets to a large extent.

We first analyzed commonly reported indicators in the microbiome field by evaluating alpha diversity, beta diversity, and changes in relative abundance at the genus level of the zOTU microbiome. We observed that not all indicators showed significant differences in the random effects models used to identify the alpha diversity differences. More than half of the studies found at least one significant difference among the three analyzed indicators. In addition to richness being higher in the patients with IBS compared to the healthy population, other alpha diversity indices were generally lower in the patients with IBS. Significant differences in beta diversity between the patients with IBS and controls were relatively more frequent and prominent in the overall dataset, with five out of seven studies showing significant differences. These results suggest distinct microbial communities within the IBS population, while also suggesting that microbial diversity is not the discriminating factor. Regardless of the high or low alpha diversity, the results of previous studies based on the same dataset are consistent with our findings (Liu et al., 2021). Therefore, the alpha diversity differences observed in some studies might have been the result of variables other than IBS status, although these variables are not yet known.

We used meta-analysis to extract bacterial genera at the genus level for modeling. Overall, the random forest model based on the PRJNA682378 dataset performed the best at the species-genus level (AUC = 0.8459), while the modeling results based on the KO features performed the best using the PRJNA1011519 dataset (AUC = 0.8676). The model based on the PRJNA682378 dataset also showed good results (AUC = 0.8113). However, superior results could not be obtained when individually examining specific studies. In light of this, we applied transfer learning to enhance the predictive power of our models. Considering the results from the different studies, we selected the PRJNA682378 model as the suitable candidate for transfer. Transfer learning was applied at the genus and pathway levels for the different studies. After transfer learning, the majority of the study results showed improvement. However, the results of the individual studies did not improve; they were essentially the same as before transfer learning. Overall, the use of transfer learning can improve model performance.

The abundance of *Ruminococcaceae*, *Anaerostipes*, and *Christensenellaceae* was increased in the patients with IBS, while tyrosine metabolism, vitamin B6, and phenethylamine were clustered in the patients with IBS. *Ruminococcaceae* and *Christensenellaceae* were negatively correlated with vitamin B6 metabolism in the patients with IBS. Tyrosine metabolism was negatively correlated with *Ruminococcaceae*. Indeed, there are some conflicting results regarding *Christensenellaceae*. For example, Kamp et al. included 67 women with IBS and 46 healthy women and used 16S rRNA gene sequencing for bacterial identification. They found that the abundance of *Christensenellaceae* R-7 group, *Collinsella*, *Ruminococcaceae* UCG-002, *Ruminococcaceae* UCG-005, and *Ruminococcaceae* UCG-014 in the IBS group was lower than in the HC group (Kamp et al., 2024). However, another study by Villanueva-Millan et al. found that in patients with IBS-C (irritable bowel syndrome with constipation), the main hydrogen producers were *Ruminococcaceae* and *Christensenellaceae* (Villanueva-Millan et al., 2022). It is because of these uncertainties that we collected datasets from multiple studies to conduct a meta-analysis for an objective and systematic evaluation. Indeed, due to the inherent heterogeneity of the IBS disease itself, it would be meaningful to collect more studies within a subtype of IBS in the future to further explore the role of specific microbes.

There are several limitations to our study. Firstly, we were limited to publicly available data, which often lack sufficient clinical information to evaluate alternative hypotheses. In the absence of this information, we could not determine whether the inconsistency between the studies merely reflected sampling biases in the selected studies. For example, some studies may only recruit individuals with more severe forms of IBS, whose microbiota may differ from other patients with IBS. In addition, the impact of the IBS subtypes on the microbiota of the patients with IBS could not be accurately concluded, particularly regarding their influence on patient diversity. Furthermore, due to our limitations, our study only utilized 16S data and could not capture classification ability at a non-genus level. Further multi-omics research is needed to quantify the abundance of microbial species and their effects on proteins and metabolites.

## Data Availability

The original contributions presented in the study are included in the article/Supplementary material, further inquiries can be directed to the corresponding authors.
