# Supplementary material for: Meta-analysis of gut microbiota alterations in patients with irritable bowel syndrome
Source: Front Microbiol. 2024 Dec 24;15:1492349. doi: 10.3389/fmicb.2024.1492349 (PMC11703917; doi:10.3389/fmicb.2024.1492349)
Supplement: Supplementary file 1 [file Data_Sheet_1.PDF]

Supplementary material

Table S1. The Wilcoxon test results of the alpha diversity metrics between the IBS and control groups.

Control median	IBS median	pvalue	measures	study
455.000	389.000	2.119E-04	Richness	PRJNA268708
4.257	4.074	0.060	Shannon	PRJNA268708
0.695	0.689	0.508	J	PRJNA268708
319.500	307.500	0.401	Richness	PRJEB11419USA
3.580	3.460	0.401	Shannon	PRJEB11419USA
0.623	0.600	0.575	J	PRJEB11419USA
374.000	339.500	0.014	Richness	PRJEB11419UK
3.752	3.580	0.349	Shannon	PRJEB11419UK
0.626	0.621	0.815	J	PRJEB11419UK
629.000	677.000	0.442	Richness	PRJNA604466
4.621	4.488	0.525	Shannon	PRJNA604466
0.715	0.702	0.181	J	PRJNA604466
272.000	270.000	0.710	Richness	PRJNA544721
3.744	3.536	0.264	Shannon	PRJNA544721
0.664	0.638	0.264	J	PRJNA544721
1165.500	1191.000	0.369	Richness	PRJNA682378
5.976	5.972	0.679	Shannon	PRJNA682378
0.850	0.848	0.679	J	PRJNA682378
161.000	151.000	0.869	Richness	PRJNA1011519
2.812	2.757	0.621	Shannon	PRJNA1011519
0.565	0.553	0.621	J	PRJNA1011519

Table S2. The PERMANOVA results of the Bray-Curtis distances between the IBS and control groups.

Study	Fstat	R2	Pvalue	Group
PRJNA268708	2.046751	0.010713351	0.006	IBS-control
PRJEB11419USA	1.261489	0.006206236	0.176	IBS-control
PRJEB11419UK	2.164543	0.007408645	0.01	IBS-control
PRJNA604466	1.882105	0.035590587	0.008	IBS-control
PRJNA544721	2.340446	0.018236234	0.001	IBS-control
PRJNA682378	1.057546	0.007142802	0.005	IBS-control
PRJNA1011519	4.281	0.028	0.001	IBS-control

Table S3. Statistical analysis of the microbial community in two groups.

Genus or pathway	IBS. mean	IBS. sd	healthy. mean	healt hy.sd	Significant differences
g__Eubacterium_coprostanoligenes_group	0.88	1.79	1.06	1.46	0.18
g__Eubacterium_hallii_group	0.69	1.77	0.56	1.39	-0.13
g__Eubacterium_ventriosum_group	0.16	0.59	0.18	0.43	0.01
g__Allisonella	0.01	0.07	0.04	0.32	0.02
g__Alloprevotella	0.39	1.75	0.31	1.18	-0.08

g__Anaerostipes	0.67	1.34	0.69	1.31	0.02
g__Barnesiella	0.34	0.64	0.43	0.75	0.09
g__Christensenellaceae_R7_group	1.27	2.58	1.63	2.51	0.36
g__Clostridium_sensu_stricto_1	0.44	1.76	0.34	1.08	-0.10
g__Coprobacter	0.04	0.28	0.03	0.06	-0.01
g__Coprococcus_3	0.16	0.27	0.20	0.31	0.03
g__Faecalitalea	0.02	0.08	0.01	0.04	-0.01
g__Holdemanella	0.27	0.86	0.32	1.01	0.06
g__Lachnospiraceae_FCS020_group	0.04	0.06	0.05	0.07	0.01
g__Lachnospiraceae_ND3007_group	0.15	0.21	0.21	0.31	0.07
g__Lachnospiraceae_UCG004	0.05	0.12	0.06	0.12	0.01
g__Lachnospiraceae_UCG008	0.21	0.43	0.32	1.33	0.11
g__Paraprevotella	0.36	1.09	0.41	0.99	0.05
g__Prevotella_7	0.19	2.23	0.39	2.16	0.20
g__Ruminococcaceae_NK4A214_group	0.34	0.78	0.59	1.04	0.25
g__Ruminococcaceae_UCG002	1.49	2.10	2.53	3.79	1.04
g__Ruminococcaceae_UCG005	0.46	0.74	0.69	1.32	0.24
g__Ruminococcaceae_UCG014	1.39	3.15	1.95	3.11	0.56
g__Ruminococcus_2	1.11	2.34	1.20	2.35	0.08
g__un_o_Mollicutes_RF39	0.33	0.92	0.70	1.53	0.37
g__un_o_Rhodospirillales	0.12	0.40	0.13	0.46	0.02
Tyrosine metabolism	0.27	0.06	0.27	0.06	0.00
Phenylalanine metabolism	0.34	0.11	0.33	0.11	0.00
Selenocompound metabolism	1.06	0.08	1.06	0.06	-0.01
D Arginine and D ornithine metabolism	0.16	0.18	0.14	0.15	-0.02
Inositol phosphate metabolism	0.21	0.05	0.20	0.05	-0.01
Aminobenzoate degradation	0.12	0.07	0.11	0.07	0.00
Carbon fixation pathways in prokaryotes	1.08	0.13	1.08	0.10	0.00
Vitamin B6 metabolism	1.16	0.14	1.16	0.14	0.00
Nitrogen metabolism	0.65	0.10	0.65	0.10	0.00
Spliceosome	0.00	0.00	0.00	0.00	0.00
Peroxisome	0.23	0.05	0.22	0.05	-0.01
Vasopressin regulated water reabsorption	0.00	0.00	0.00	0.00	0.00

Table S4. Pre-transfer and post-transfer results at the functional prediction level.

Dataset	Transfer Auc	Auc
PRJEB11419UK	0.52672	0.54077
PRJEB11419USA	0.55795	0.58466
PRJNA1011519	0.81818	0.86759
PRJNA268708	**0.67696**	0.62033
PRJNA544721	**0.61034**	0.51379
PRJNA604466	**0.74359**	0.53846

* Transfer AUC indicates that the AUC after transfer, and the thickening indicates that the result is improved after transfer learning.

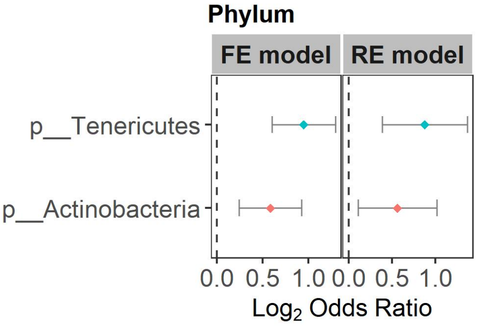

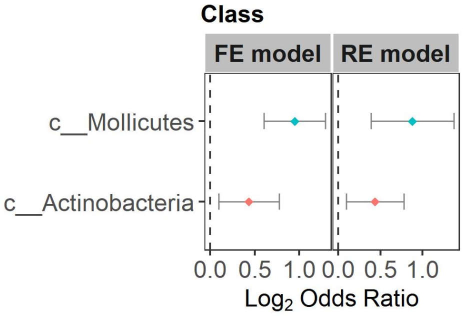

A **B**

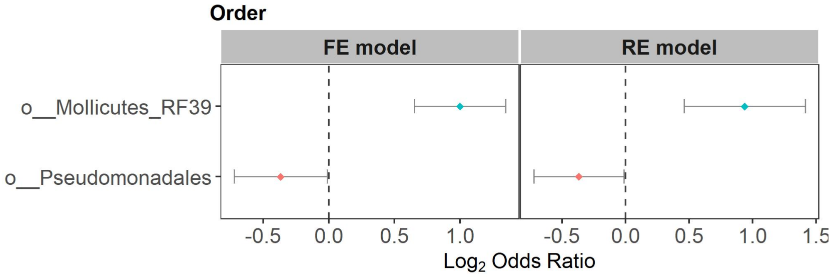

C

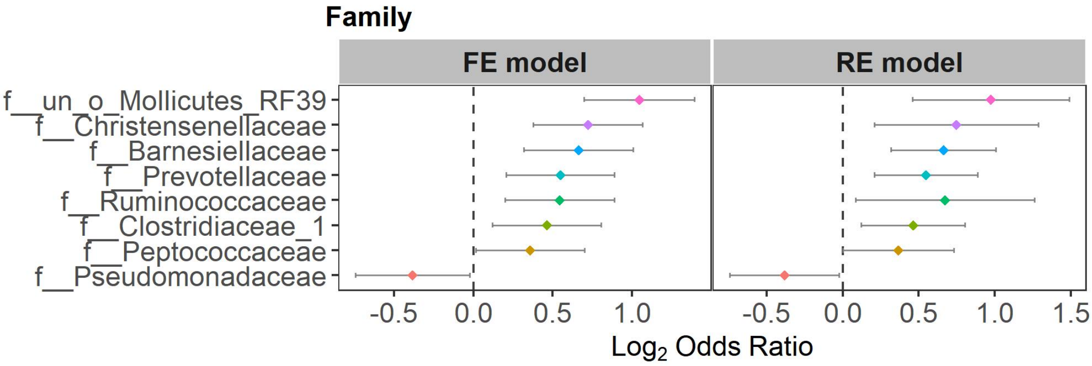

D

Figure S1. Random forest plots of (**A**) Phyla, (**B**) Classes, (**C**) Orders and (**D**) Families with significant ORs.

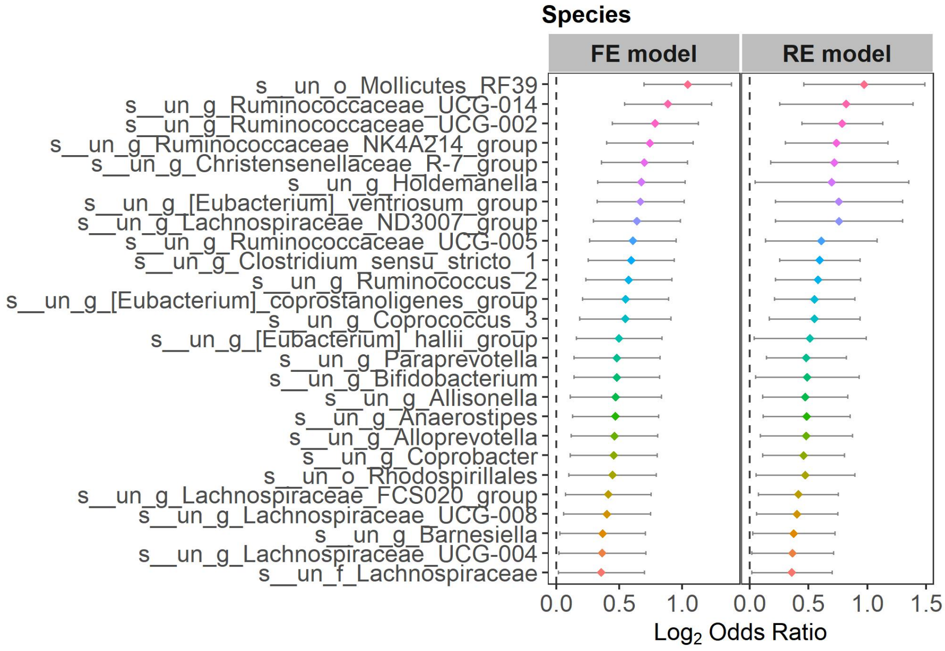

Figure S2. Random forest plots of Species with significant ORs.

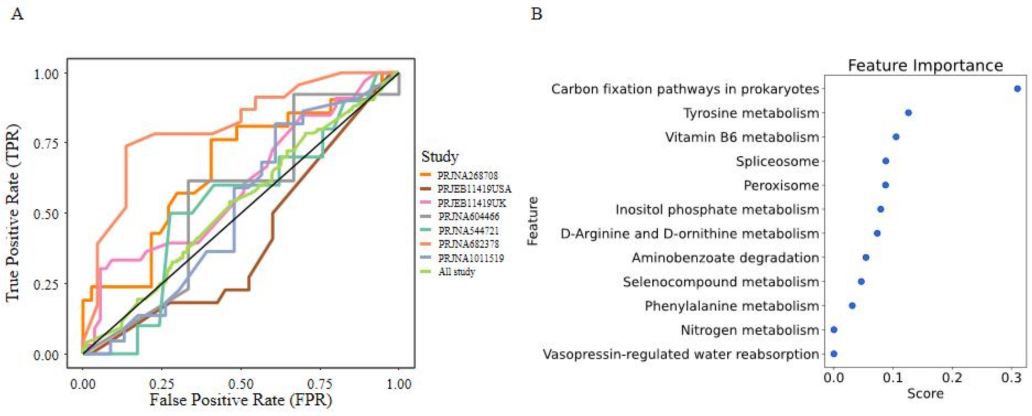

Figure S3. Performance of RF model: (A) AUC curve at functional prediction level. The specific AUC value of the training set and test set is shown in Table 3; (B) KO level feature importance.

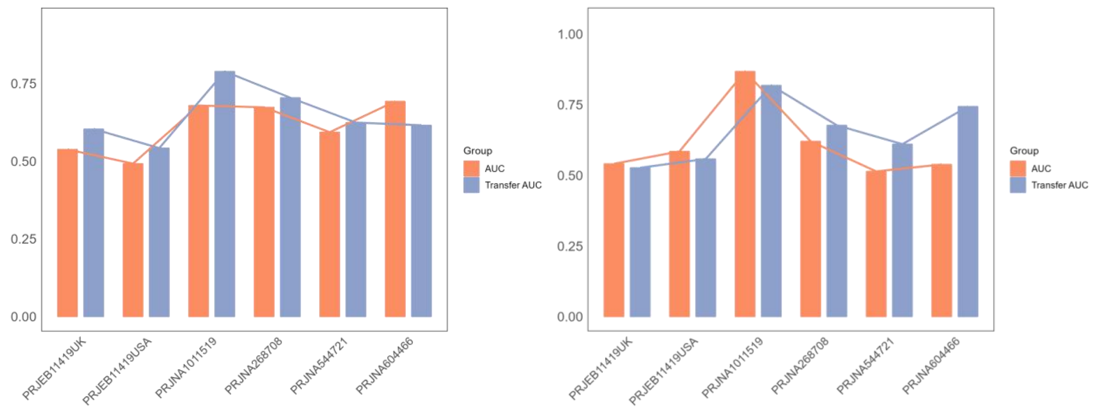

A

B

Figure S4. Comparison of results before and after transfer: (**A**) The specific AUC of the training set and test set at the genus level, and the specific AUC value is shown in Table 4; (**A**) The specific AUC of the training set and test set at functional prediction level, and the specific AUC value is shown in Table S4.
